# Relationship Between Plasma Leptin Levels and Airflow Limitation in the Small and Medium Airways in Young Adults

**DOI:** 10.3390/jcm14051624

**Published:** 2025-02-27

**Authors:** Rodrigo Muñoz-Cofré, Edgardo Rojas-Mancilla, Pablo A. Lizana, Máximo Escobar-Cabello, Claudio García-Herrera, Daniel Conei, Fernando Valenzuela-Aedo, Francisco Javier Soto-Rodríguez, Mariano del Sol

**Affiliations:** 1 Ciencias Morfológicas, Universidad de La Frontera, Temuco 4811230, Chile; fernando.valenzuela@ufrontera.cl (F.V.-A.); mariano.delsol@ufrontera.cl (M.d.S.); 2 Escuela de Kinesiología, Facultad de Salud, Universidad Santo Tomás, Manuel Rodríguez 060, Temuco 4801076, Chile; 3 Unidad de Diagnóstico Laboratorio Clínico, Instituto Oncológico, Fundación Arturo López Pérez, Santiago 7500691, Chile; edgardo.rojas@falp.org; 4 Escuela de Terapia Ocupacional, Facultad de Salud y Ciencias Sociales, Universidad de Las Américas, Santiago 7500975, Chile; 5 Laboratory of Epidemiology and Morphological Sciences, Instituto de Biología, Pontificia Universidad Católica de Valparaíso, Valparaíso 2373223, Chile; pablo.lizana@pucv.cl; 6 Laboratorio de Función Disfunción Ventilatoria, Departamento de Kinesiología, Universidad Católica del Maule, Talca 3530000, Chile; maxfescobar@gmail.com; 7 Departamento de Ingeniería Mecánica, Universidad de Santiago de Chile, Santiago 9170022, Chile; claudio.garcia@usach.cl; 8 Departamento de Procesos Terapéuticos, Facultad de Ciencias de la Salud, Universidad Católica de Temuco, Temuco 4780000, Chile; dconei@uct.cl; 9 Departamento de Ciencias de la Rehabilitación, Facultad de Medicina, Universidad de La Frontera, Temuco 4811230, Chile; francisto.soto@ufrontera.cl; 10 Programa de Doctorado en Medicina Clínica y Salud Pública, Universidad de Granada, 18071 Granada, Spain; 11 Centro de Excelencia en Estudios Morfológicos y Quirúrgicos (CEMyQ), Facultad de Medicina, Universidad de La Frontera, Temuco 4811230, Chile

**Keywords:** body composition, leptin, airflow, airway, young adult

## Abstract

**Background/Objectives**: To determine the relationship between plasma leptin levels and airflow limitation (AFL) in the small and medium airways of young adults according to their body composition (BC). **Methods**: To determine AFL, the following measurements were taken: forced expiratory volume in the first second (FEV_1_), forced expiratory flow between 25–75% (FEF_25–75%),_ airway resistance (Raw), and specific airway resistance (sRaw). The measured BC variables were body mass index (BMI), body fat percentage (%BF), trunk fat percentage (TF%), and fat-free mass (FFM). Plasma leptin was measured using the ELISA technique. **Results**: In total, 83 participants (40 male and 43 female) aged 21.55 ± 2.08 years were evaluated. Leptin was significantly higher in women than men (*p* < 0.01). When relating leptin to lung function variables, FEV_1_ and FEF25–75% showed a significant inverse relationship (*p* < 0.01), and Raw and sRaw showed a direct and significant relationship (*p* < 0.01). Female participants with a BF% and leptin higher than their p75 were observed to have a higher risk of increased sRaw (OR = 1.0; OR = 1.15, respectively), regardless of age, and participants with an FFM% higher than their p75 had a lower risk of increased sRaw (OR = 0.71), regardless of gender and age. **Conclusions**: There is an inverse relationship between FEV_1_, FEF_25–75%_, and a direct relationship between Raw and sRaw with leptin. For female participants with a higher BF%, higher FFM%, and leptin, the risk of developing Raw and sRaw was found to be modified.

## 1. Introduction

Breathing is an essential function for survival; therefore, changes related to lung function may decrease the quality of life and performance of daily activities [1]. Several mechanisms have been proposed that may affect the mechanics of the respiratory system, most notably body composition (BC) [2,3]. Specifically, the effects of muscle mass, lean body mass, and fat mass on lung function have been studied, showing that there is a close relationship between BC and forced vital capacity (FVC) and forced expiratory volume in the first second (FEV_1_) [4]. Therefore, changes in BC would affect the medium-sized airway [5,6], a phenomenon that does not ensure the same behavior in small airways. In a previous study, it was found that in young people between 20 and 29 years of age, airflow limitation was reversible for 5.3% and irreversible for 2% of the population [7]. In this context, excessive weight gain or loss has been negatively associated with alterations in FVC and FEV_1_ [2,4]. In addition, increased waist circumference, waist-to-hip ratio, and body fat mass percentage (BF%) have been associated with decreased lung function [2].

In more detail, an excessive accumulation of fat mass alters the relationship between the lungs, chest wall, and diaphragm, decreasing lung volume, and negatively impacting the cross-sectional diameter of the airways [4]. This effect could be mediated, in part, by an inflammatory process. In this context, the endocrine and paracrine action of adipose tissue produces cytokines and bioactive mediators, promoting a proinflammatory state [8,9]. This is associated with pulmonary hypoplasia and bronchial hyperresponsiveness [10]. Leptin regulates a wide range of systemic functions, from satiety to inflammation, and several studies have shown that leptin and its ObR receptor are expressed in adipose tissue but also in other tissues, such as the lungs [11], where leptin has been implicated in large airway inflammatory processes [11,12].

The presence of leptin receptors in the bronchi, alveolar tissue, and airway smooth muscle leads to questions about its role in airway diameter [12]. Leptin can decrease airway diameter, regardless of body mass index (BMI), by acting on the central nervous system to inhibit parasympathetic outflow to the bronchial tree, which may explain excessive bronchoconstriction in normal-weight and obese patients [13]. Although its involvement in chronic respiratory pathologies has been studied, the link between serum leptin levels and lung function in healthy subjects remains to be described [14].

Regarding airflow limitation (AFL), FEV_1_ is one of the most easily accessible variables, being the most studied. However, including other variables such as forced expiratory flow between 25–75% (FEF_25–75%_) and airway resistance (Raw) in subjects without a history of respiratory disease would be useful, since they provide information on the small airway [15] for the early diagnosis of respiratory problems, leading to the prevention of their chronic phase [12,16]. This study aims to determine the relationship between plasma leptin levels and AFL in the small and medium airways of young adults according to their BC.

## 2. Materials and Methods

### 2.1. Participants

This study was approved by the Ethics Committee of the University of Santiago de Chile (14/2020) per The Code of Ethics of the World Medical Association (Declaration of Helsinki) for experiments involving humans. The study aims were explained orally to all participants at the beginning and written informed consent was obtained. To calculate the sample size, the statistical program eNe 3.0 was used, considering the study by Rodríguez et al., with a sample of 57 participants and a specific airway resistance (sRaw) of 3.8 ± 1.03 cmH_2_O*s. A power of 80% and a significance level of 5% were used [17]. This resulted in 36 participants of each gender considering a 10% drop-out rate. The inclusion criteria required participants to be above 18 years of age, to exhibit no signs of chronic or acute respiratory disease, and to have normal spirometric readings (FEV_1_ > 80%, predicted). Participants with smoking habits or morphological alterations of the thorax or spine, and without comorbidities or anti-inflammatory treatments were excluded. Sampling was conducted in March 2020. All study participants were tested in a single morning session at the Pulmonary Function Laboratory of the Universidad Católica del Maule, Chile.

### 2.2. Anthropometry

Height was measured with a SECA^®^ anthropometer (model 220, Hamburg, Germany), obtaining the distance from the ground to the vertex in meters, with the participant standing with heels together and feet at a 45° angle. The heels, buttocks, back, and occipital region were in contact with the surface of the anthropometer. This was done at maximum inspiration, keeping the head in the Frankfurt plane. Body mass was determined by measuring with a SECA^®^ scale (model 840, Hamburg, Germany) [18]. The BMI was obtained by the mathematical operation of dividing weight (kg) by height (m) squared (kg/m^2^). A BMI < 18.5 kg/m^2^ was considered underweight, normal weight between 18.5 kg/m^2^ and 25 kg/m^2^, overweight between 25 kg/m^2^ and 30 kg/m^2^, and obesity with a BMI greater than 30 kg/m^2^ [19].

### 2.3. Body Composition

A bioelectrical impedance scale (TANITA MC-780 MA, Tanita Corporation, Tokyo, Japan) was used to evaluate BC. For the measurement, each participant was asked not to carry metallic objects, not to have drunk alcohol 48 h before the assessment, not to have performed intense exercise 12 h before the assessment, not to have eaten or drunk, especially caffeine or diuretics, 4 h before the assessment, and they were asked to urinate before the assessment [20]. The variables analyzed were weight, BF%, visceral fat index (VFI), trunk fat mass percentage (TF%), and free fat mass percentage (FFM%).

### 2.4. Lung Function

**Spirometry:** A Mediagraphics body plethysmograph (Platinum Elite DL^®^, St. Paul, Minnesota, USA) was used. According to the acceptability and reproducibility criteria standardized by the American Thoracic Society (ATS), the highest FVC value of the three attempts was recorded. The variables used were FEV_1_ and FEF_25–75%_. All participants were required to have normal spirometry readings (FEV_1_ and FVC > 80% predicted) according to ATS guidelines [21].

**Ventilatory volumes:** Tests to assess lung volumes were performed on a Mediagraphics body plethysmograph (Platinum Elite DL^®^, St. Paul, Minnesota, USA). The cabin was closed, and four ventilations at tidal volume were indicated. The participant was instructed to ‘pant gently’, attempting to move volumes of 50–60 mL while blocking their cheeks with the ends of their fingers to avoid fluctuating mouth pressure. The panting frequency had to be close to 60 per minute (1 Hz). The attendant activated the shutter for 2 to 3 s, after which a maximal inspiration and then an exhalation up to residual volume (RV) was indicated. Measurements were performed according to ATS standards [22]. The variables used were Raw and sRaw.

**Maximal inspiratory and expiratory pressures (MIP-MEP):** To assess MIP, the individual was directed to execute a maximal exhalation, after which the pneumotachograph was occluded, and a maximal inspiration against the closed valve was requested. To assess MEP, the individual was directed to execute a maximal inhalation, after which the pneumotachograph was occluded, and a maximal expiration against the closed valve was requested. The best test was selected from a minimum of three acceptable and reproducible maneuvers in both tests according to ATS standards [23].

### 2.5. Blood Plasma Sample

Blood samples were obtained in the morning by venipuncture, following 8 h of fasting. Blood was dropped in a glass tube with ethylenediaminetetraacetic acid (EDTA), centrifuged at 1500× *g* for 5 min, and the plasma (supernatant) was transferred to a cryotube to be frozen at −80 °C until analysis.

### 2.6. Leptin

Plasma leptin (human leptin ELISA kit, ab99978, Abcam, Cambridge, MA, USA) was measured using the ELISA technique according to the manufacturer’s instructions. Briefly, the first and second plasma samples were thawed slowly until they reached room temperature, in the same way as the reagents. Samples were added to the wells covered by anti-IL-6 and anti-leptin antibodies, the wells were washed, and the secondary antibody was added. The streptavidin was added and then washed. The substrate was added and incubated at room temperature to generate the color. Finally, the reaction was stopped, and the reading was carried out on a spectrophotometer (Tecan, Infinite, Grödig, Austria) at 450 nm. A calibration curve was constructed, and the concentration of IL-6 and leptin in each sample was calculated.

### 2.7. Statistical Analysis

Descriptive statistics were used to summarize the data. The statistical program STATA 15 (StataCorp. 2017. Stata Statistical Software: Release 15. College Station, TX, USA: StataCorp LP) was used. Data normality was assessed using the Shapiro–Wilk test. To assess differences among anthropometric, BC, and lung function variables between men and women, the Student’s *t*-test or the Mann–Whitney U-test for independent samples was used. Pearson’s or Spearman’s R test was conducted based on the data distribution to determine the correlation between leptin, BC, and lung function. Binary logistic regression analyses were performed to estimate the association between leptin/BC and lung function variables. The following percentiles were used as cut-off points to dichotomize the data: 75th percentile (p75) for the variable leptin, BF%, obese BMI (OBMI), VFI, TBF%, Raw, and sRaw, and the 25th percentile (p25) for FEV_1_ and FEF_25–75%_. This means the leptin levels for men are >644 pg/mL and are >1583 pg/mL for women. In BC, the BF% for men is >25%, and for women is >35%. The OBMI for men and women is >30 kg/m^2^, and the TBF% for men is >28.5% and for women is >30.45%. For lung function, a Raw >1.16 cmH_2_O/L/s in men and >1.54 cmH_2_O/L/s in women is considered high. A sRaw in men >3.77 cmH_2_O*s and women >4.47 cmH_2_O*s is considered high. An FEV_1_ of <4.04 L/s in men and <3.09 L/s in women is considered low. A FEF_25–75%_ in men <3.96 L/s and in women <3.02 L/s is considered low. All binary logistic regressions were adjusted for age and gender, with the event of interest being female participants. To verify the model fit, the Hosmer–Lemeshow test was applied. The level of significance was set at *p* < 0.05.

## 3. Results

The anthropometric and BC characteristics of the participants are shown in Table 1, of which ten participants are obese. The sample revealed significant differences in the height variable by gender (*p* < 0.01). BF% (30.34 ± 5.70) was significantly higher in women than men (*p* < 0.01). FFM% was significantly higher in men (*p* < 0.01) than women (*p* < 0.01). Leptin was significantly higher in women than men (*p* < 0.01).

The variables airway flow, volume, and pressure were significantly higher in men than in women (*p* < 0.01, see Table 2). Raw (1.17 ± 0.52 cmH_2_O/L/s) was significantly higher in women (*p* < 0.01) than in men (Table 2).

When relating leptin to lung function variables, FEV_1_, and FEF_25–75%_ showed a significant inverse relationship (*p* < 0.01). In addition, Raw and sRaw showed a direct and significant relationship (*p* < 0.01). A significant inverse relationship was also observed between leptin and FFM% (r = −0.73; *p* < 0.01), and a direct and significant relationship between leptin and BF% as well as between leptin and TF% (*p* < 0.01; Table 3).

In the associations of leptin, lung function, and BC, models were adjusted for gender and age (Table 4). For Raw, participants with a BF% above their p75 were found to be at higher risk of an increased Raw (OR = 1.4), regardless of gender and age. Similarly, participants with a TBF% above their p75 have a higher risk of having an increased Raw (OR = 1.3), irrespective of gender and age. However, participants with an FFM% higher than their p75 had a lower risk of increased sRaw (OR = 0.71), regardless of gender and age (Table 4). Concerning sRaw, female participants with BF% and leptin higher than their p75 were observed to have a higher risk of increased sRaw (OR = 1.0; OR = 1.15, respectively), regardless of age (Table 4). Female participants with a TBF% and leptin above their p75 are also at risk of increased sRaw (OR = 1.0; OR = 1.1, respectively), regardless of age and gender (Table 4). In contrast, in women, increased FFM% decreased the risk of increased sRaw, regardless of high leptin (OR = 0.8) (Table 4).

## 4. Discussion

This study examined the correlation between leptin and AFL in small and medium-sized airways by evaluating leptin levels alongside FEV_1_, FEF_25–75%_, Raw, and sRaw. The main results were the inverse relationship between FEV_1_ and FEF_25–75%_ with leptin and the direct relationship between Raw and sRaw with leptin. Also, female participants with a higher BF% and leptin were found to be at higher risk of increased Raw and sRaw. Finally, women with a higher FFM% have a lower risk of developing Raw and sRaw.

Leptin is a peptide hormone secreted mainly by adipose tissue and can modulate food intake, body weight, and reproductive and immunological functions. Its levels increase proportionally to fat mass. It has been shown that gender and age determine its level of expression, being significantly higher in women, adults, and older adults [24]. Recently, a study evaluating plasma proteins linked to cardiovascular risk identified correlations between specific proteins, including leptin, and reduced FEV_1_ and FVC while maintaining a stable FEV_1_/FVC ratio, a pattern consistent with restrictive physiology [25]. Hickson et al. (2011) studied the association between serum leptin and spirometric lung function measurements in an African-American population with a mean age of 54.3 ± 12.4 years, where 62.7% of the sample were women. Their results indicated that men with a high serum leptin concentration had significantly lower lung function [12]. Additionally, there was a significant inverse association between serum leptin and lung function in obese women. Although the sample studied here is a younger age range and Amerindian ethnicity, we also observed an inverse relationship between serum leptin levels with FEV_1_ and FEF_25–75%_. In addition to this, we found a direct relationship between serum leptin levels and Raw and sRaw [26]. Thus, leptin levels could be an indicator linked to AFL regardless of age and ethnicity.

In 2019, Zaw et al. studied the relationship between anthropometric indices, serum leptin levels, and lung function in healthy subjects aged 18–45. Their results indicated that median and interquartile ranges of leptin were significantly higher in obese subjects than in non-obese subjects (5.8 [3.5–9.1] ng/mL vs. 1.9 [1.1–3.1] ng/mL, *p* < 0.001). They also noted that serum leptin levels had a significant negative relationship with FVC, FEV_1_/FVC, FEF_25–75%_, and PEF [14]. These results are consistent with those of the present investigation; however, the proposed methodology allowed for incorporating variables that characterize BC in more detail, such as BF%, TBF%, and FFM, thus describing more fully the relationship between lung function and BC.

Parastesh et al. (2020) studied the effects of an aerobic training program on lung function and serum leptin levels in obese men aged 47.03 ± 3.36 years. Their results showed that normal-weight subjects had a significant decrease in leptin levels (*p* < 0.01) and a notable increase in lung function (*p* < 0.016) compared to obese subjects. In addition, leptin and obesity have a significant inverse relationship with FVC and FEV_1_ (*p* ≤ 0.05) [27]. This may suggest that fluctuations in leptin levels are sensitive to alterations in body composition and the subsequent impact on lung function.

The role of inflammation in the airway is well known from the study of various lung diseases, where anti-inflammatory treatments prevent a decrease in lung function. In this sense, McNeill et al. (2021) studied the link between inflammatory pathways and impaired lung function, as measured by their response to cardiopulmonary exercise, in 695 obese individuals. The researchers observed that leptin was associated with lower FEV_1_, FVC, TLC, and diffusing capacity of the lungs for carbon monoxide (DLCO), suggesting a restrictive respiratory pattern [28]. The findings of the present investigation confirm that obesity affects the airways ‘causing an incipient obstructive pattern’. This is to be expected, considering that obesity, and specifically increased visceral adiposity, promotes systemic inflammation [11,26,27].

Lower expression levels of leptin and ObR in the bronchial epithelial cells of patients with severe asthma, compared to healthy individuals, have been documented, demonstrating an inverse relationship with airway remodeling [29]. In addition, significantly higher leptin levels have been demonstrated in people with asthma compared to controls (*p* < 0.01), regardless of gender and age [30]. In support of this, a meta-analysis of 13 studies with 3642 patients found that an asthma diagnosis was associated with higher leptin levels in both adults and children; however, this association disappeared in some studies when the results were adjusted for body weight [31]. In this context, one of the exclusion criteria for the present study was the presence of respiratory pathologies, which would reinforce the relationship between leptin, inflammation, and airway dysfunction in the absence of chronic respiratory disorders.

It has been suggested that leptin may modulate bronchial diameter, which may explain bronchoconstriction in obese subjects, increasing its diameter in response to transforming growth factor beta-1 activity, decreasing the anti-fibrotic activity of PPAR-gamma [13,32] and increasing the activation of pro-fibrotic genes [33]. In this regard, an alteration of Raw, marked by an increase in pro-inflammatory parameters, such as leptin, IL-1 beta, IL-8, and TNF-alpha, and a decrease in anti-inflammatory parameters, such as adiponectin and IL-10, has been seen in older adults with metabolic syndrome [34,35]. This background reinforces that ventilatory function is affected by a state of obesity and its immune response. Future research should encompass pro- and anti-inflammatory pathways in people with no chronic respiratory history to augment the findings of this study.

Fat tissue is the most variable component of BC [36,37]. The metabolic and mechanical consequences of regional fat distribution have been reported and are particularly important in clinical assessment and therapeutic approaches [38,39]. Obesity has increased alarmingly in underdeveloped countries [40]. One of the most commonly used measures to assess obesity is BMI. However, it is not a measure that distinguishes the components of the human body (fat mass, muscle mass, bone mass) [41,42]. In this context, it should be noted that in this study, no associations were observed by BMI; nevertheless, associations were identified among the variables constituting BC, indicating that BMI assessment may hinder the early detection of respiratory issues.

This study has limitations that must be stated. The reported results only applied to the group evaluated and cannot be generalized. However, the agreements with other authors render the data found in Raw and sRaw relevant.

## 5. Conclusions

In conclusion, there is an inverse relationship between FEV_1_, FEF_25–75%_ with leptin, and a direct relationship between Raw and sRaw with leptin. Also, female participants with a higher BF% and leptin were found to be at higher risk of increased Raw and sRaw. Finally, women with a higher FFM% have a lower risk of developing Raw and sRaw.

## Figures and Tables

**Table 1:** Anthropometric and body composition characteristics of the total sample by gender.

	Total Sample	Male	Female	*p* Value
*n* (%)	83 (100)	40 (48.19)	43 (51.81)	-
Age (years)	21.55 ± 2.08	21.93 ± 2.73	21.85 ± 2.17	0.27 ^MW^
Weight (Kg)	68.26 ± 11.76	74.26 ± 15.78	70.18 ± 12.68	<0.66 ^t^
Height (m)	1.65 ± 0.09	1.71 ± 2.70	1.65 ± 0.09	<0.01 ^t^
Leptin (pg/mL)	820.91 ± 614.5	423.9 ± 339.4	1230.0 ± 489.90	<0.01 ^t^
BMI (Kg/m^2^)	25.15 ± 4.32	25.35 ± 4.94	25.33 ± 4.42	0.79 ^MW^
BF%	24.79 ± 8.66	19.39 ± 7.60	30.34 ± 5.70	<0.01 ^t^
TF%	23.89 ± 7.84	22.29 ± 8.60	25.53 ± 6.71	0.07 ^t^
FFM%	75.21 ± 8.66	80.59 ± 7.60	69.66 ± 5.70	<0.0001 ^MW^

Results are presented as mean ± standard deviation. kg: kilograms; m: meters; pg/mL: picograms per milliliter; BMI: body mass index (kilograms divided by the square of the height in meters); BF%: body fat percentage; TF%: trunk fat percentage; FFM%: fat-free mass percentage; MW: Mann–Whitney; ^t^, Student’s t.

**Table 2:** Lung function characteristics of the total sample by gender.

	Total Sample	Male	Female	*p* Value
FVC (L)	4.52 ± 0.95	5.25 ± 0.66	3.78 ± 0.54	<0.01 ^t^
FEV_1_ (L/s)	3.86 ± 0.76	4.43 ± 0.53	3.28 ± 0.47	<0.01 ^t^
FEF_25–75%_ (L/s)	4.13 ± 0.94	4.61 ± 0.93	3.63 ± 0.68	<0.01 ^t^
PEF (L/s)	8.08 ± 1.72	9.38 ± 1.28	6.75 ± 0.90	<0.01 ^MW^
SVC (L)	4.06 ± 0.89	4.74 ± 0.73	3.50 ± 0.60	<0.01 ^t^
ERV (L)	1.33 ± 0.43	1.55 ± 0.46	1.15 ± 0.31	<0.01 ^t^
IC (L)	2.72 ± 0.75	3.19 ± 0.79	2.32 ± 0.50	<0.01 ^t^
RV (L)	1.92 ± 0.71	2.26 ± 0.81	1.56 ± 0.48	<0.01 ^t^
TLC (L)	5.89 ± 1.40	6.81 ± 1.41	5.03 ± 0.75	<0.01 ^MW^
MIP (-cmH_2_O)	102.72 ± 33.04	117.4 ± 34.32	87.59 ± 23.09	<0.01 ^t^
MEP (cmH_2_O)	99.76 ± 26.98	112.8 ± 25.75	86.38 ± 21.30	<0.01 ^t^
Raw (cmH_2_O/L/s)	1.00 ± 0.51	0.83 ± 0.45	1.17 ± 0.52	0.03 ^MW^
sRaw (cmH_2_O*s)	3.35 ± 1.33	3.24 ± 1.36	3.46 ± 1.31	0.40 ^MW^

FVC: forced vital capacity; FEV_1_: volume that has been exhaled at the end of the first second of forced expiration; FEF_25–75%_: forced expiratory flow 25–75%; PEF: Peak expiratory flow; L: liters; s: seconds; MIP: maximum inspiratory pressure; cmH_2_O: centimeters of water; MEP: maximum expiratory pressure; SVC: slow vitality capacity; ERV: expiratory reserve volume; IC: inspiratory capacity; RV: residual volume; TLC: total lung capacity; Raw: airway resistance; sRaw: specific airway resistance; cmH_2_O/L/s: centimeters of water divided by liters divided by seconds; cmH_2_O*s: centimeters of water per second; MW: Mann–Whitney; ^t^: t-Student. Median ± standard deviation.

**Table 3:** Relationship between anthropometric and body composition variables and indicators of airway obstruction in the total sample.

	FEV_1_ (L/s)	FEF_25–75%_ (L/s)	Raw (cmH_2_O/L/s)	sRaw (cmH_2_O*s)	Leptin (pg/mL)	BMI (Kg/m^2^)	BF (%)	TF (%)	FFM (%)
FEV_1_ (L/s)	-	-	-	-	-	-	-	-	-
FEF_25–75%_ (L/s)	0.80 <0.01	-	-	-	-	-	-	-	-
Raw (cmH_2_O/L/s)	−0.34 <0.01	−0.37 <0.01	-	-	-	-	-	-	-
sRaw cmH_2_O*s)	−0.09 0.47	−0.21 0.08	0.87 <0.01	-	-	-	-	-	-
Leptin (pg/mL)	−0.54 <0.01	−0.47 <0.01	0.56 <0.01	0.46 <0.01	-	-	-	-	-
BMI (Kg/m^2^)	−0.15 0.23	−0.20 0.10	0.23 0.06	0.15 0.22	0.18 0.13	-	-	-	-
BF (%)	−0.46 <0.01	−0.31 0.01	0.68 <0.01	0.53 <0.01	0.73 <0.01	0.25 0.04	-	-	-
TF (%)	−0.11 0.38	−0.09 0.47	0.64 <0.01	0.61 <0.01	0.50 <0.01	0.21 0.07	0.87 <0.01	-	-
FFM (%)	0.46 <0.01	0.31 0.01	−0.68 <0.01	−0.53 <0.01	−0.73 <0.01	−0.25 0.04	−1.00 <0.01	−0.87 <0.01	-

FEV_1_: volume exhaled at the end of the first second of forced expiration; FEF_25–75%_: forced expiratory flow 25–75%; L/s: liters/seconds; Raw: airway resistance; sRaw: specific airway resistance; cmH_2_O/L/s: centimeters of water divided by liters divided by seconds; BMI: body mass index (kilograms divided by the square of the height in meters); BF%: body fat percentage; TF%: trunk fat percentage; FFM%: fat-free mass percentage; cmH_2_O*s: centimeters of water per second.

**Table 4:** Logistic regressions for the association among pulmonary function measured and body composition adjusted by gender and age.

	FEV_1_(L/s)		FEF _25–75%_ (L/s)		Raw(cmH_2_O/L/s)		sRaw(cmH_2_O*s)	
	OR [95%CI]	*p*	OR [95%CI]	*p*	OR [95%CI]	*p*	OR [95%CI]	*p*
Gender (female)	0.55 [0.13–2.38]	0.42	2.37 [0.51–7.18]	0.33	0.23 [0.01–5.37]	0.357	0.096 [0.011–0.783]	0.029
Age (years)	1.04 [0.79–1.39]	0.78	0.97 [0.76–1.24]	0.79	1.47 [0.81–2.65]	0.202	1.053 [0.733–1.513]	0.778
Leptin	1.06 [0.74–10.16]	0.13	1.17 [0.34–4.07]	0.80	2.21 [0.23–21.13]	0.491	1.002 [1.001–1.004]	0.001
Obese BMI (≥30 kg/m^2^)	2.74 [0.02–1.49]	0.11	0.28 [0.05–1.49]	0.13	4.86 [0.05–45.46]	0.162	2.264 [0.491–10.448]	0.295
Hosmer-Lemeshow	>0.05		>0.05		>0.05		>0.05	
Gender (female)	0.75 [0.15–3.78]	0.79	2.37 [0.57–9.87]	0.24	0.27 [0.01–6.45]	0.417	0.058 [0.007–0.484]	0.008
Age	1.01 [0.77–1.34]	0.92	0.97 [0.76–1.24]	0.83	1.24 [0.73–2.1]	0.420	1.031 [0.711–1.495]	0.869
Leptin	3.09 [0.72–13.34]	0.13	1.60 [0.40–6.49]	0.51	0.86 [0.07–11.2]	0.907	1.001 [1.000–1.003]	0.017
Fat mass %	0.33 [0.06–1.87]	0.21	0.26 [0.05–1.30]	0.10	1.41 [1.01–1.95]	0.042	1.154 [1.015–1.312]	0.028
Hosmer-Lemeshow	>0.05		>0.05		>0.05		>0.05	
Gender (female)	0.45 [0.09–2.21]	0.33	1.82 [0.48–6.92]	0.38	2.90 [0.06–143.795]	0.593	0.170 [0.025–1.130]	0.067
Age (years)	1.01 [0.76–1.34]	0.93	0.97 [0.76–1.24]	0.81	1.21 [0.74–1.98]	0.447	1.023 [0.709–1.478]	0.900
Leptin	1.97 [0.52–7.40]	0.32	0.99 [0.28–3.53]	0.99	1.25 [0.11–14.19]	0.857	1.002 [1.000–1.003]	0.011
Trunk fat %	0.84 [0.21–3.29]	0.81	0.77 [0.22–2.70]	0.69	1.36 [1.09–1.84]	0.044	1.148 [1.019–1.295]	0.023
Hosmer-Lemeshow	>0.05		>0.05		>0.05		>0.05	
Gender (female)	0.75 [0.15–3.79]	0.73	2.37 [0.57–9.85]	0.24	0.27 [0.01–6.47]	0.418	0.058 [0.007–0.485]	0.009
Age (years)	1.01 [0.77–1.34]	0.92	0.97 [0.76–1.24]	0.83	1.24 [0.73–2.11]	0.421	1.031 [0.711–1.495]	0.870
Leptin	1.00 [0.10–1.00]	0.09	1.00 [0.99–1.00]	0.43	0.99 [0.99–1.00]	0.406	1.001 [1.000–1.003]	0.017
Fat-free mass %	1.07 [0.96–1.19]	0.20	1.04 [0.95–1.14]	0.39	0.71 [0.51–0.99]	0.042	0.866 [0.762–0.984]	0.028
Hosmer-Lemeshow	>0.05		>0.05		>0.05		>0.05	

BMI: body mass index (kilograms divided by the square of the height in meters); OR: odds ratio; CI: confidence interval; FEV_1_: volume exhaled at the end of the first second of forced expiration; FEF_25–75%_: forced expiratory flow 25–75%; Raw: airway resistance; sRaw: specific airway resistance; cmH_2_O/L/s: centimeters of water divided by liters divided by seconds; cmH_2_O*s: centimeters of water per second; 75 p of Raw male >1.16; female: >1.54; sRaw, male >3.77; female >4.47; 25 p FEV1: male <4.04; female <3.09; FEF_25–75%_: male <3.96; female <3.02.

## Data Availability

The data used in this research is available. Please send a request to Dr. Rodrigo Muñoz-Cofré.

## Abbreviations

The following abbreviations are used in this manuscript:

BC	Body composition
FVC	Forced vital capacity
FEV_1_	Forced expiratory volume in the first second
BF%	Body fat mass percentage
BMI	Body mass index
AFL	Airflow limitation
FEF_25–75%_	Forced expiratory flow between 25–75%
Raw	Airway resistance
sRaw	Specific airway resistance
VFI	Visceral fat index
TF%	Trunk fat mass percentage
FFM%	Free fat mass percentage
RV	Residual volume
MIP	Maximum inspiratory pressure
MEP	Maximum expiratory pressure
DLCO	Diffusing capacity of the lungs for carbon monoxide
FEF	Forced expiratory flow
PEF	Peak expiratory flow
SVC	Slow vitality capacity
ERV	Expiratory reserve volume
IC	Inspiratory capacity
TLC	Total lung capacity
